# Pakistan amid WHO (World Health Organization) red listed countries and mental health risk for nurses – A cross-sectional study

**DOI:** 10.12669/pjms.41.12.12196

**Published:** 2025-12

**Authors:** Azeem Kaleem, Changaiz Dil Essa

**Affiliations:** 1 Azeem Kaleem, Assistant Professor, Lahore School of Nursing, The University of Lahore, Lahore, Pakistan; 2 Changaiz Dil Essa, Principal, Mustafa Kamal Institute of Nursing and Medical Sciences, Vehari, Pakistan

**Keywords:** Mental health, Nurses, WHO code of practice, World Health Organization

## Abstract

**Objective:**

The study aimed to investigate the mental health of nurses in Pakistan who are eligible to get employment in the UK but still struggling to do so because of WHO (World Health Organization) code of practice.

**Methodology:**

This cross sectional study was conducted between October, 2024 to November, 2024. Data was collected from 72 participants via Warwick-Edinburgh Mental Well-being Questionnaire in addition to an open-ended question. Structured questionnaire was analyzed via descriptive analysis whereas, manifest content analysis was utilized for open-ended question.

**Results:**

Majority (63.9%) of the participants were female nurses as compared to male nurses who were 36.1%. The analysis revealed that 48.6% of the participants were categorized to have very low mental well-being on Warwick-Edinburgh Mental Well-being Scale. The content analysis revealed the accounts of mental frustration, financial loss and other emotional concerns.

**Conclusion:**

There is a dire need for WHO policy makers, national healthcare regulatory bodies, and educational system to address the concern of nurses belong to amber/red listed countries in order to promote their mental well-being.

## INTRODUCTION

There are 106,473 registered nurses in Pakistan according to the statistical report of PNC (Pakistan Nursing Council) which seems to fall short for the 241.49 million population of the country. Despite the shortage of healthcare personnel and resources in Pakistan, nurses seek employment in abroad due to variety of reasons, such as higher compensation packages, favorable environment for future medical facilities, fulfillment of human rights, and family satisfaction.^1^ For the pursuance of the aforementioned requirements, it is believed that the United Kingdom is relevant and practical for nurses of Pakistan. However, the recent report of the code of practice and amber list of countries by World Health Organization (WHO)^2^ seems to cause lasting turmoil among nurses who find less opportunities in Pakistan to excel both professionally and personally.

To elaborate, Pakistani nurses must go through a number of procedures to meet the requirements for UK Nursing and Midwifery Council approval. These processes mainly include OET (Occupational English Test) or IELTS (International English Language Testing System) and CBT (Computer base test) which cost a lot of financial burden for nurses living in developing countries like Pakistan. It is used to take several months for nurses to go to the UK after passing these examinations, but the NHS UK has been vigilant in recruiting Pakistani staff nurses since the code of practice report. It goes without saying that nurses who put in a lot of time and effort in clearing the aforementioned exams, run the danger of experiencing a mental health crisis if the outcome is impractical due to WHO code of practice.

It is reported that failure in pursuit of important goals affects negatively in greater proportion than less important goals.^3^ Similarly, repeated failures to achieve important goals may lead to chronic distress and anxiety disorders.^4^ Conclusively, nurses in Pakistan who put in financial and mental investment towards achieving their goal of international employment in the UK face repetitive number of rejections from NHS hospitals, are at eventual risk of mental health disorders.

The study aimed to investigate the mental status of nurses who have passed their OET/IELTS, CBT, and are still struggling to get employment in the UK.

## METHODOLOGY

This descriptive cross-sectional study was conducted during the period of October 2024 to November 2024. The study was conducted through a general survey and achieved objectives.

### Ethical approval:

The ethical approval was sought from Research Ethical Committee of The University of Lahore (Ref# REC-UOL-/520/08/24; dated September 24, 2025). Moreover, the researcher ensured the implementation of basic research ethical principles during the completion of research study i.e., the participants were ensured confidentiality, privacy, and anonymity of the data.

The study included participants who have completed the following criteria, OET/IELTS, CBT, passed for more than six months and applied for NHS/UK caring homes jobs. The total number of respondents were 72, both male and female nurses. These participants were recruited through convenient sampling. To elaborate, WhatsApp groups and YouTube channel were utilized to recruit the study participants because of their easiest accessibility, recruit ability and researcher’s feasibility. These platforms were provided a Google Form generated link consisting of numerous components i.e., informed consent, demographic characteristics, and questionnaire. The informed consent elaborated the benefits/risks of the research study. Second component was based on the demographic characteristics of the participants i.e., gender, age, education, passed time period of OET/IELTS, CBT, and job hunting on the portals of NHS/UK nursing home care. The last component included the questionnaire of Warwick - Edinburgh Mental Well-being Scale which intends to measure the positive aspects of an individual’s mental well-being.

The scale with the Cronbach alpha value of 0.89- 0.91^5^ provides a monitoring report to evaluate the status of an individual’s mental well-being and an assessment measure to employ initiatives if needed, to promote the mental well-being of an individual. The scale consists of 14 items with a minimum value of 14 and maximum value is 70. The interpretation of the scale is based on the total marks obtained by the individual which includes 14-40 (very low), 41-44 (below average), 45-59 (average), and 60-70 (above average).^6^ Furthermore, the researcher added an open-ended question (How Pakistan being red listed country in WHO’s code of practice has affected your mental health?) in order to have a comprehensive understanding of the subject matter.

### Statistical analysis:

The study included all participants based on the eligibility criteria. The data were analyzed using descriptive statistics which include percentage and frequency. Moreover, the overall results were also compared with the interpretation suggested by the Warwick- Edinburgh Mental Well-being scale. Statistical analysis was performed through SPSS version 26 for quantitative descriptive analysis. In addition, manifest content analysis was conducted to analyze the open-ended question. This type of content analysis focuses on visible and obvious words described in the answers given by the participants.^7^ The text was thoroughly examined and aimed to explore the noticeable descriptions given by majority of the participants and therefore presented in the findings of the research study.

## RESULTS

In total, 72 participants completed the survey. Majority (63.9%) of the participants were female nurses as compared to male nurses who were 36.1%. The demographic characteristics including age and education are illustrated in Table-I.

**Table-I:** Age and Education of the participants.

Age	Frequency	Percentage
18-25	6	8.3
26-35	46	63.9
36-45	16	22.2
46-55	4	5.6
Total	72	100.0
Education		Frequency
Diploma		13
Bachelor		37
Master		22
Total		72

Furthermore, on the Warwick and Edinburgh Mental Well-Being scale, majority of the participants’ scores fell into the very low (48.6%) category group, suggesting a concerningly low level of mental well-being that requires attention. Other category groups included average, below average and above average with their percentages 26.4%, 13.9%, and 11.1% respectively. The percentages of the participants for the individual items of the questions are illustrated in Table-II.

**Table-II:** Percentage of the participants on Warwick- Edinburgh Mental Well-being Scale.

Item	None of the time	Rarely	Some of the time	Often	All of the time
I have been feeling optimistic about the future		13.9	37.5	23.6	25.0
I have been feeling useful		22.2	30.6	19.4	27.8
I have been feeling relaxed	5.6	34.7	37.5	16.7	5.6
I have been feeling interested in other people	2.8	34.7	47.2	15.3	
I have had energy to spare	2.8	27.8	31.9	18.1	19.4
I have been dealing with problem well		23.6	26.4	27.8	22.2
I have been thinking clearly		27.8	29.2	20.8	22.2
I have been feeling good about myself	5.6	18.1	36.1	18.1	22.2
I have been feeling close to other people	2.8	29.2	40.3	19.4	8.3
I have been feeling confident	2.8	18.1	33.3	29.2	16.7
I have been able to make my mind about things	2.8	25.0	41.7	19.4	11.1
I have been feeling loved	2.8	36.1	27.8	8.3	25.0
I have been feeling interested in new things	2.8	26.4	34.7	19.4	16.7
I have been feeling cheerful	2.8	50.0	18.1	9.7	19.4

According to the results, it was revealed that 37.5% of the participants occasionally ponder about their bright future. Majority of the participants reported that they felt useful, relaxed, energetic, and interested in people on infrequent basis with their respective percentages of 30.6%, 37.5%, 31.9%. It was also reported that 22.2% of the participants were all clear about their thinking processes, feeling good about themselves, and were able to execute effective problem solving without any stressor. In addition, 29.2%, 18.1%, 25.0%, 36.1%, 26.4%, and 50.0% of the participants respectively accounted for a rare feeling of closeness to other people, lack of confidence, inability to make mind about things, inability to feel loved, unwillingness to try new things, and cheerlessness.

**Fig.1:**
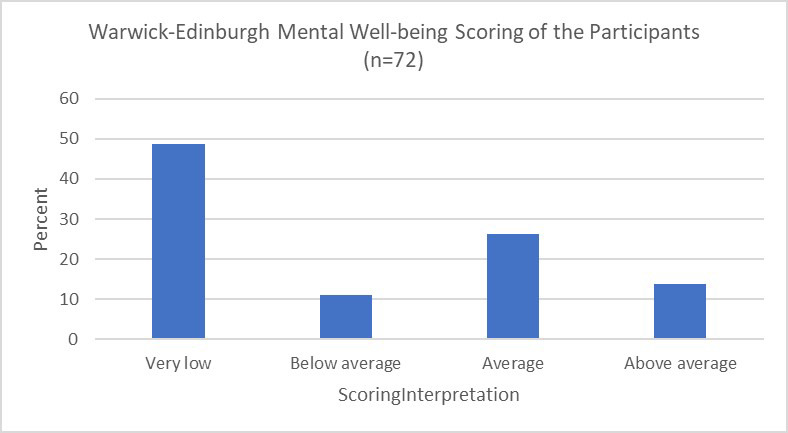
Warwick-Edinburgh Mental Well-being Scoring of the Participants (n=72).

This illuminates a significant proportion of participants being affected by mental health crisis and therefore needs healthcare assistance. To elaborate, a high number of participants (48.6%) fall in the category termed as very low on Warwik-Edinburgh Mental Well-being scale i.e., 14-40 score which is indicative of the procurement of less positive aspects of mental-health among majority of the participants’ lives (as illustrated in Fig.1).

### Content Analysis:

An additional aspect of the questionnaire was open-ended question, aimed to be analyzed via manifest content analysis approach. This method seeks to draw attention to the terms that appear most frequently in the participants’ text. Out of 72 participants, only 23 (31.9%) responses were provided by the participants in this component of data collection. Upon analysis, it was identified that majority of the participants (73.9%) accounted for demotivation after the bill passed in WHO’s code of practice. As one of the participants stated, *“When I started this process, I was very much excited, I passed my OET and CBT in first attempt but after that I heard about the ban on international vacancies and Pakistan being in the red list. It stressed me out. I started feeling demotivated”* (PT 12). Similarly, another participant reported, *“I feel depressed and (feel) low most of the time. I have low confidence now, (I am) de-motivated and it (WHO’s code of practice) has affected my decision making”* (PT 44). In relation to decision making, seven (30.4%) participants revealed that the code of practice has influenced their future decision making in an ineffective manner. One participant articulated, *“Sometimes it troubles my mind about deciding the future goals but I still hope that they will lift the ban from Pakistan”* (PT 17). 60.8% (fourteen) of the participants clearly expressed that they are mentally distressed about the UK not hiring nurses from Pakistan due to WHO’s code of practice. One participant narrated, *“It is very frustrating for me to cope up as I have cleared those challenging exams and still not having any option to get a job in the UK. The delay and no hiring from my country made me feeling mentally distressed”* (PT 23). Another participant reported, “*When I started my OET journey, I was very optimistic about my future and after passing CBT, when I started applying for jobs, I became unsuccessful even after several months. I started feeling disappointed, frustrated, and mentally distressed. Thinking of the energy, time, and the money I spent for all these processes, I often feel very depressed*” (PT 56). Likewise, another participant shared, *“(Pakistan) being red listed in WHO’s code of practice, not only my mental health is affected but also my whole aspect of life”* (PT 03). Few (34.7%) of the participants were hopeful about getting the ban removed by WHO in the near future whereas, some (43.4%) were anxious about the dark future of Pakistani nurses. As it was stated by one of the participants, *“Now a day Pakistan current situation by the ban of WHO’s code of practice has disturbed our mental health, but I am hopeful for better future”* (PT 10). On the contrary, one participant reported, *“It is not only affecting us but also our next generations. Future is dark for our children due to this code. Our families’ hopes are going to be ruined. It is very inhumane to make restrictions like these and putting (stigma) on skilled people”* (PT07).

Conclusively, majority of the participants accounted for mental frustration, financial loss, personal and professional stagnation in growth and development with mixed emotional future concerns. These aspects need to be addressed on immediate basis via practical measures taken on both national and international level.

## DISCUSSION

The study aimed to investigate the mental health status of overseas Pakistani nurses who had passed their OET/IELTS, CBT and are struggling to find employment in the United Kingdom due to the code of practice placed by World Health Organization. The study results reveal that majority of nurses fall under the category of very low mental well-being. To elaborate, challenge faced by Pakistani nurses has affected their confidence, interest in social interactions & personal relationships, and self-integration within community in terms of usefulness. This finding is consistent with previous literature which insinuates the critical association of mental health with employment. The literature describes that stable employment enroute the nurturing of self-confidence, societal, and inter-personal involvement whereas unstable employment leads to long term negative impact on mental health 8,9

The study also identified that inability to achieve the goal of international employment in the UK has caused confusion, mental frustration, sense of exhaustion, and emotional instability among majority of Pakistani nurses which is consistent with one of the previous study conducted in drivers who were found in mental frustration and emotional behaviors when triggered by achievement obstacles and unpredictable experiences.^10^ Similary, another study reveals that goal failure predicts apprehension to failing again in the future among individuals.^11^ Interestingly, Pakistani nurses who met the UK Nursing and Midwifery Council’s eligibility requirements were unable to fulfill their aspirations of working abroad, which led to feelings of powerlessness, annoyance, and anxiety - all of which are signs of mental distress. Moreover, the study identified that the effect of the code of practice set by WHO caused majority of the Pakistani nurses to report cheerlessness, lack of productivity, and self-criticism. This finding is consistent with previous study conducted in medical students who upon their goal failure experienced extreme emotional breakdown which involves aggression, unhappiness, and sense of inferiority.^12^

Notably, it was discovered that the WHO code of practice incident had an impact on Pakistani nurses’ reports of weak decision-making and problem-solving skills as well as the sustainability of their future aspirations. This finding is congruent with the previous studies aimed at investigating the lived experiences of unemployed individuals. The studies found that the individuals felt directionless with no positive future outcomes.^13^-^15^ It is believed that in the current study, Pakistani nurses are thought to have an existing job in their nation; nonetheless, the desire for an international job is significantly more privileged than that of a domestic one, therefore, the latter can be considered as unemployment in this case.

Conclusively, the current study findings suggest an alarming situation among Pakistani nurses who seek international employment in the UK and experience mental health crisis due to the code of practice applied by WHO. It is noted that with such mental health condition and continued practice at local healthcare system, Pakistani nurse seems to provide inadequate patientcare and insufficient healthcare quality outcomes which is debilitating for overall healthcare system and therefore demands an immediate response from Pakistani healthcare system. Evidently, the healthcare system of Pakistan contributes to the mental health of patients but neglects to address the mental health of healthcare professionals, particularly nurses, who serve as the primary caregivers of patients. Nonetheless, this study has brought attention to the employment dissatisfaction that nurses encounter in Pakistan which aspire nurses to opt for overseas employment and failing to do so may influence their mental health which requires an action plan to be sought by healthcare policy makers for quality healthcare delivery.

Notably, there are several initiatives that can be taken to prevent mental distress among Pakistani nurses. Above all, having a sense of safety and security in one’s living environment is a basic right. The national government must address the scarcity of Pakistani nurses in terms of future security, job satisfaction, and employee retention. Moreover, local authorities must develop quality nursing educational institutions to meet the challenge of healthcare staff shortage. This will not only strengthen the national healthcare system but also enable the revision of WHO code of practice and thereby economic growth. It is also noted that WHO needs to acknowledge the fact that the shortage of healthcare professionals is not a new phenomenon, it is a global concern and is being dealt by both developed and developing countries like Pakistan.

Remarkably, if Pakistan is short of nurses, then OEC (Overseas Employment Corporation), a public sector recruitment agency of Pakistan^16^ would have halted the process of migrating female nurses to gulf countries in greater proportion for employment purposes. Comparably, male nurses are less frequently recruited by OEC and consequently prioritize the route of the UK as an employment goal. Hence, male nurses are presumed to be more vulnerable to experience mental health issues than female nurses. Similarly, The UK’s Nursing and Midwifery Council could authorize the deterrence of nurses from amber list nations for initial registration for CBT. This action will facilitate the decrease of financial load on the nurses belonging to amber/red list countries. In addition, it will help Pakistani nurses to forego repeated rejections by the UK employers, futile paperwork commitments and thereby, mental anguish.

Undoubtedly, Pakistani nursing students, upon completion of their degrees, strive to live abroad, therefore, there is a need to educate our nursing students to be resilient in successful goal achievement. According to the literature, approach goal strategy proves to be effective in times of unexpected failures as opposed to avoidance goal strategy. The former is success oriented while the latter is based on tasks accomplishments to limit negative outcomes. It is reported that individuals with approach goals are better able to identify the situations which require complete detachment from failure than people with avoidance goals.^17^ Similarly individuals from variety of occupations evidently achieve their desired career outcomes through the utilization of goal setting theory.^18^-^20^

### Strengths of the study:

A major strength of the study is that it is among the first to investigate the impact of WHO code of practice employment guidelines on the mental health of caregivers belonging to red list/amber list countries particularly in Pakistan. This implies that the WHO should review the code of practice or implement strategies to address the concern of mental distress among nurses from such countries. Furthermore, the study explores a thorough perspective on the subject matter and contributes to the body of knowledge in a comprehensive manner by providing the data in both quantitative and qualitative way which is another strength of the study.

### Limitations:

The study has small sample size which could affect generalizability of the study. However, the study provides an insight into further research studies and interventions aimed at promoting the mental well-being of caregivers who belong to red/amber list countries as per WHO code of practice.

## CONCLUSION

There is a dire need to address this concern of global mental health risk for nurses belong to red and amber list countries like Pakistan. The WHO policy makers, national healthcare regulatory bodies, and educational system ought to meet the challenges faced by such nurses in order to promote their mental well-being.
